# Compatible Solutes Prevent Lung Inflammation and Reduction in CFTR Induced by Combustion-Derived Nanoparticles in Human and Rodent Experimental Systems

**DOI:** 10.3390/ijms26199487

**Published:** 2025-09-28

**Authors:** Tim Spannbrucker, Klaus Unfried, Tamara Hornstein

**Affiliations:** IUF—Leibniz Research Institute for Environmental Medicine, Auf’m Hennekamp 50, 40225 Duesseldorf, Germany

**Keywords:** cystic fibrosis transmembrane conductance regulator, carbon nanoparticles, carbon black, neutrophilic granulocytes, neutrophil-like HL-60 cells, ectoine, 5-hydroxyectoine, Nγ-acetyl-L-2,4-diaminobutyric acid, neutrophil apoptosis, air pollution

## Abstract

The compatible solute ectoine is known to attenuate inflammatory effects in the airways after exposure to combustion-derived nanoparticles. Pro-inflammatory signaling in epithelial cells, as well as antiapoptotic mechanisms in neutrophilic granulocytes, both triggered by particles, are reduced by this substance. Here we investigated the preventive potential in airway inflammation of additional compounds originating from the ectoine metabolism, Nγ-acetyl-L-2,4-diaminobutyric acid (NADA), and 5-hydroxyectoine in a mouse model and in human neutrophilic granulocytes. Furthermore, effects of these molecules on the reduction in cystic fibrosis transmembrane conductance regulator (CFTR), as an additional pathogenic endpoint of nanoparticle exposure, were investigated. All three solutes exhibited beneficial effects at the level of inflammatory cells in lung lavages from exposed mice. The decrease in CFTR in lung tissue of exposed mice was mitigated by the substances. In primary human neutrophils and in neutrophilic differentiated HL-60 cells, the delay of apoptosis rates after particle exposure was effectively abolished. The decline in CFTR from the cytoplasmic membrane in neutrophilic cells was also counteracted by the compatible solutes. The data identify both NADA and 5-hydroxyectoine as additional substances for molecular prevention of airway effects of environmental particles. Furthermore, the reduction in CFTR might be a relevant finding for patients suffering from impaired function of this ion channel.

## 1. Introduction

Compatible solutes are a heterogeneous group of biomolecules, consisting of amino acids and their derivates, small carbohydrates, or methylamines and methylsulfonium solutes, which allow cells to maintain their life functions under extreme conditions [1]. Particularly extremophilic microorganisms produce these compounds in order to cope with high salt concentrations or high temperatures in their natural environment. As osmotic active substances, these compounds are able to counteract osmotic stress without disturbing the hydration layer of macromolecules like proteins, nucleic acids, or membrane structures [2]. Due to the principle of preferential exclusion, they force these biomolecules into their thermodynamically most stable conformation [1]. Interactions between membranes and membrane-coupled proteins, like membrane receptors, are stabilized and cellular functions are maintained under extreme conditions [3,4,5]. These properties characterize them as ideal candidates for biotechnical and medical applications [2].

The most frequently used compatible solute is ectoine (1,4,5,6-tetrahydro-2-methyl-pyrimidinecarboxylic acid), which was first isolated from the halophilic bacterium *Ectothiorhodospira halochloris* [6]. Meanwhile the substance is produced on an industrial scale, mostly by a process called “bacterial milking”, from *Halomonas elongata* [7]. In addition to some biotechnical applications, ectoine is mostly used in cosmetics and in medical devices aiming to protect epithelia against environmental hazards, including cremes against inflammatory skin diseases and dermatitis, eye drops and nasal sprays against symptoms of dryness and allergy, and inhalation solutions to prevent and treat airway diseases [8].

Some of the molecular mechanisms of the beneficial effects of ectoine are well investigated and understood. In human keratinocytes in the presence of ectoine, ceramide production, as a feature pro-inflammatory membrane-coupled signaling after UV-irradiation, is reduced [9,10]. Similar effects were observed when lung epithelial cells were exposed to environmental model particles (carbon nanoparticles). The induction of reactive oxygen species by this environmental stressor leads to a degradation of membrane lipids and a reorganization of membrane signaling units (lipid rafts), which are involved in several pro-inflammatory effects, including the upregulation of IL-8, the main chemokine responsible for neutrophilic lung inflammation [11,12]. The preventive effect of ectoine against this pathogenic endpoint could therefore be well documented in a couple of animal experiments, in which neutrophilic lung inflammation was induced by exposure to carbon nanoparticles [12,13]. In the presence of 1 mM ectoine, neutrophil influx was reduced by approx. 30%. Moreover, time-course experiments showed that neutrophilic inflammation is more rapidly resolved in the presence of ectoine [14]. This effect appears to be mediated by modulation of the life span of neutrophilic granulocytes. Natural apoptosis is a major regulator of neutrophilic lung inflammation. Under pro-inflammatory conditions, including an oxidative microenvironment, natural apoptosis rates are reduced and the life span of these cells is prolonged leading to an exacerbation of inflammation [15]. This life-extending mechanism is triggered by the intracellular oxidative stress after carbon nanoparticle exposure and, to some extent, counteracted by the stabilizing effect of ectoine. The prevention of reduced apoptosis rates by ectoine was demonstrated in experiments with animals exposed to carbon nanoparticles via intratracheal instillation and also in peripheral blood neutrophils from chronic obstructive pulmonary disease (COPD) patients and healthy volunteers exposed to the same material ex vivo [14]. These data indicate that ectoine, and some additional compatible solutes tested so far, counteract inflammatory airway inflammation at the level of induction of pro-inflammatory factors in epithelial cells and also at the level of life span regulation of inflammatory neutrophils [16]. The human relevance of these effects of such highly compatible natural product was also demonstrated in a human intervention study, in which elderly female volunteers who suffered from mild symptoms of COPD due to lifelong exposure to environmental traffic pollution were employed [17]. The inhalation of a single daily ectoine dose for 28 days led to a reduction in neutrophil numbers and nitrite in sputum compared with the placebo group (daily saline inhalation). These results, together with a couple of data sets from clinical and observational studies, suggest to use ectoine as a molecular preventive or a mild therapeutic against airway inflammation [18].

The stabilizing effects of compatible solutes may also be used to address pathogenic effects of the malfunction of cystic fibrosis transmembrane conductance regulator (CFTR). Mutations in the gene for this chloride channel affect its functionality and have severe pathological outcomes including increased mucus production in the airways. Early studies on the protein maturation and function of a specific CFTR mutation (*ΔF508*) revealed that the presence of compatible solutes during protein biosynthesis leads to proper protein folding and increased channel functionality [19]. Epithelial functionality, however, can also be affected by the loss of CFTR from the cell membrane e.g., after cigarette smoke exposure, which leads to the activation of a signaling cascade via EGFR and MAP kinases after induction of intracellular oxidative stress [20]. The same pathway is activated after exposure to carbon nanoparticles as a model of environmental combustion-derived nanoparticles [21]. In a more recent study, we were able to show that bronchial epithelial cells exposed to spark-generated pure carbon nanoparticles show decreased CFTR protein levels accompanied by a downregulation of CFTR gene expression [22]. As suggested by an in vitro study, ectoine in the presence of pharmaceutical CFTR correctors, by suppressing the EGFR cascade, may prevent the reduction in CFTR from the membrane of lung epithelial cells and therefore maintain epithelial functionality in patients bearing the *ΔF508* mutation [23].

The chloride channel CFTR is also present in the membranes of neutrophilic granulocytes. In this cell type the bulk amount of the channel is located in the membranes of phagosomes. After phagocytosis of pathogens the channels provide chloride ions and allow myeloperoxidase to generate hypochloric acid in these vesicles contributing to the oxidative inactivation of pathogens [24]. A smaller amount of CFTR is located in the cell membrane, which under inflammatory conditions is responsible for the release of chloride ions in the extracellular space. Excreted myeloperoxidase may then produce extracellular hypochloric acid and thus contribute to an oxidative microenvironment [25].

**Figure 1:**
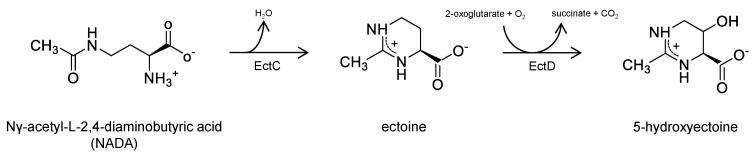
Compatible solutes of ectoine metabolism in *Halomonas elongata* [28]. Nγ-acetyl-L2,4-diaminobutyric acid is converted to ectoine by *ectoine synthase* (EctC). *Ectoine hydroxylase* (EctD) catalyzes the formation of 5-hydroxyectoine from ectoine.

As compatible solutes are well tolerated by volunteers or patients, the substance group has the potential to provide additional compounds for medical applications (Figure 1). In addition to ectoine, several prokaryotes also produce 5-hydroxyectoine mediated by the enzyme *ectoine hydroxylase* (EctD), using ectoine as a substrate [26]. As far as investigated, 5-hydroxyectoine appears to display protective and anti-inflammatory effects very similar those of its precursor molecule ectoine [27]. An additional candidate for testing is the linear precursor molecule of ectoine Nγ-acetyl-L-2,4-diaminobutyric acid (NADA) [28]. During biosynthesis NADA is converted to ectoine by *ectoine synthase* (EctC) catalyzing a cyclic condensation reaction. Earlier studies have shown that NADA but not its non-acetylated precursor *L-2,4-diaminobutyric acid* has an osmoprotective impact in diverse prokaryotes with impaired ectoine biosynthesis [29,30]. In vitro assays with purified NADA revealed that this substance, like its downstream derivates ectoine and 5-hydroxyectoine, has a beneficial impact on enzyme activity also under high-temperature conditions [31].

As additional compatible solutes might outreach the potential of ectoine in the protection or therapy of airway inflammation, we aimed to study the effects of 5-hydroxyectoine and NADA in comparison to ectoine. For that purpose we chose the mouse model, in which neutrophilic lung inflammation was induced by a single application of carbon nanoparticles (CNP; carbon black, Printex90) at 5 mg/kg of body weight in suspension by pharyngeal aspiration. The impact of increasing concentrations of compatible solutes on lung inflammation was determined by differential analyses of inflammatory cells in lung lavages after 12 h. Additionally, the effects of the compounds on delayed apoptosis as a pro-inflammatory mechanism was tested in peripheral blood neutrophils from volunteers and in HL-60 cells differentiated to primed neutrophils exposed to this material [32]. As carbon nanoparticles appear to affect cellular functions by inducing the decrease in CFTR localized in the cytoplasmic membrane, we also investigated the effect of the compatible solutes on this endpoint in both experimental systems in the lung tissue of exposed mice and in neutrophilic cells.

## 2. Results

**Figure 2:**
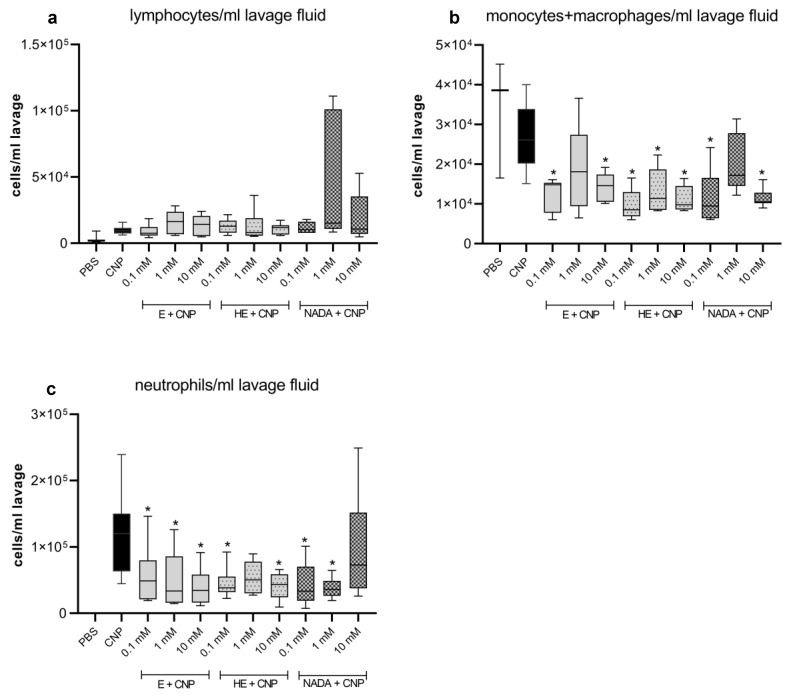
Effect of compatible solutes on carbon-nanoparticle-induced neutrophilic lung inflammation in vivo. Female C57BL/6JRj mice (*n* = 7 per group) were exposed via pharyngeal aspiration of carbon nanoparticles (CNPs) at 5 mg/kg of body weight in the presence or absence of 1 mM ectoine (E), 5-hydroxyectoine (HE), and Nγ-acetyl-L-2,4-diaminobutyric acid (NADA). Numbers of lymphocytes (**a**), monocytes/macrophages (**b**), and neutrophils (**c**) were determined in broncho-alveolar lavage 12 h after exposure of animals. Data are presented as box plots (whiskers min to max), *n* = 7; * *p* ≤ 0.05.

Carbon nanoparticles can be considered as a model of the pure carbon core of environmental nanoparticles [33]. Inhalation of this kind of materials leads to a rapid induction of neutrophilic lung inflammation, which is characteristic for such exposure [34]. We therefore developed in vivo and in vitro systems to study the cellular and molecular effects of this material. In order to investigate the effects of 5-hydroxyectoine and NADA in comparison to ectoine, we applied carbon nanoparticles (carbon black, Printex90) suspended in PBS, or the indicated concentrations of compatible solutes, as a single bolus of 5 mg/kg of body weight by pharyngeal aspiration in female C57Bl/6 mice (*n* = 7 per group). We previously observed the peak of neutrophil influx in mouse lungs after 12 h following the application of this dose of nanoparticles as a single bolus exposure [13]. Therefore, in the current experiments lung inflammation was determined at this time point by analyzing differential cell numbers in lung lavage (Figure 2). Lymphocytes were not considered as a major cell type occurring at this time point and we did not observe a significant lymphocyte increase after particle exposure (Figure 2a). Accordingly, compatible solutes had no effect on lymphocyte numbers as well. However, at the level of monocytes/macrophages and neutrophils, we observed statistically significant effects for treatment groups with compatible solutes (two-way ANOVA; *p* = 0.0001) (Figure 2b,c). Direct comparison of each group with the carbon nanoparticle group revealed the indicated differences (asterisks in Figure 2b,c). Interestingly, in this experiment we were not able to determine dose-response effects within each compatible solute (two-way ANOVA; *p* > 0.5).

**Figure 3:**
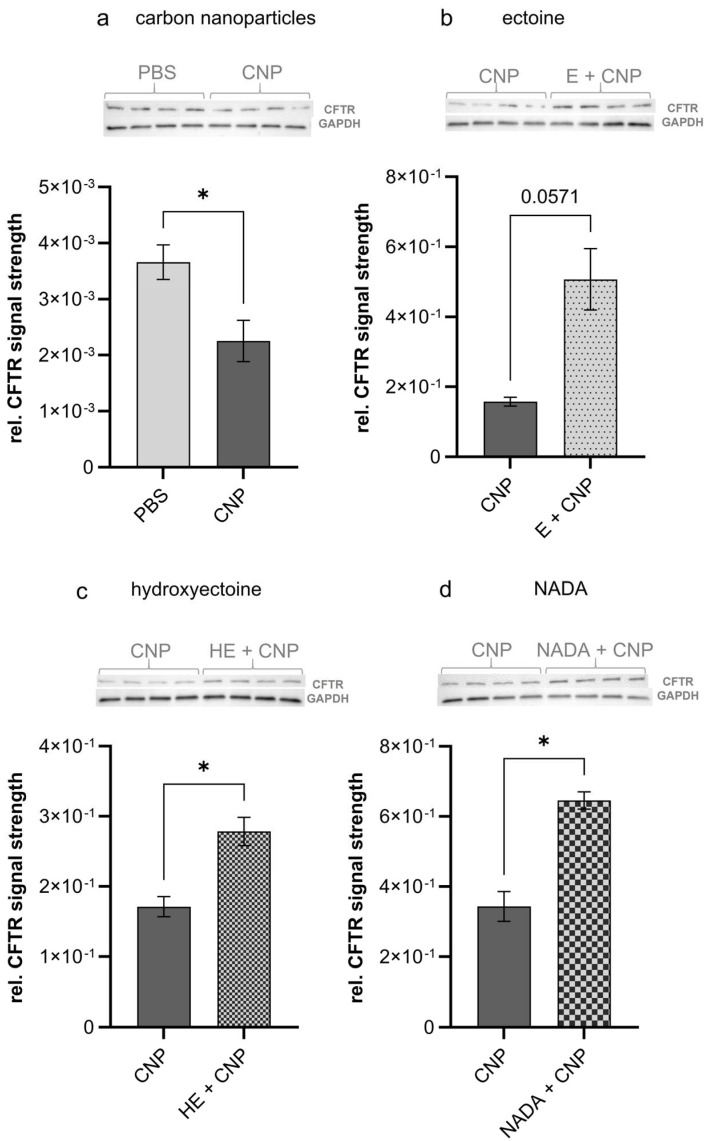
Effect of compatible solutes on carbon-nanoparticle-induced decrease in CFTR in the lung. Female C57BL/6JRj mice were exposed via pharyngeal aspiration to carbon nanoparticles (CNPs) at 5 mg/kg of body weight in the presence or absence of 1 mM ectoine, 5-hydroxyectoine (HE), and Nγ-acetyl-L-2,4-diaminobutyric acid (NADA). CFTR expression was analyzed in lung homogenates using specific antibody for Western blotting 12 h after exposure of animals. Quantitative analysis of CFTR expression in relation to GAPDH and representative Western blots are shown. Uncropped blots are shown in Supplementary Figure S1. Data are presented as mean ± SEM, *n* = 4; * *p* ≤ 0.05.

The loss of CFTR in lung epithelium may be considered as a pathogenic factor in cystic fibrosis patients but also in individuals exposed to air pollutants like cigarette smoke or carbon nanoparticles [20,22]. We therefore investigated the amount of this protein in homogenates of lung tissue from exposed mice via semiquantitative Western blot analyses (*n* = 4). The effects of compatible solutes were determined by using tissue samples of mice, which had been exposed to CNP (at 5 mg/kg of body weight) in the presence of 1 mM of each compound. In accordance with the previous in vitro findings, we now were able to demonstrate that CFTR was significantly reduced in lung tissue after short-term exposure to carbon nanoparticles (Figure 3a). Interestingly, all three compatible solutes were able to counteract these effects (Figure 3b–d).

**Figure 4:**
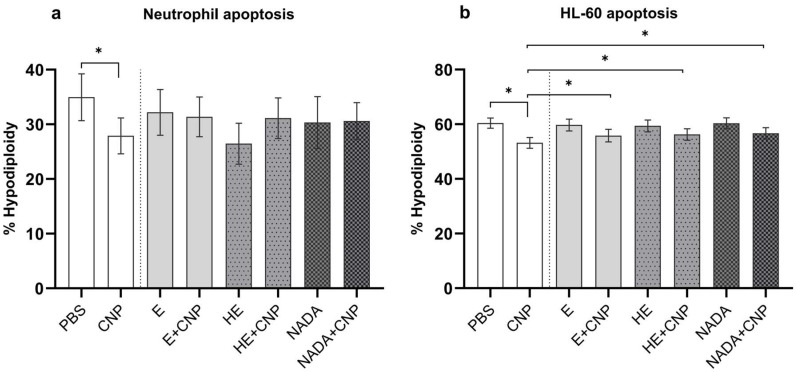
Effect of compatible solutes on carbon-nanoparticle-induced apoptosis delay in human neutrophils and differentiated HL-60 cells. Freshly isolated human peripheral blood neutrophils (**a**) and differentiated neutrophil-like HL-60 cells (**b**) at density of 2 × 10^6^ cells/mL were pretreated with 1 mM ectoine (E), 5-hydroxyectoine (HE), and Nγ-acetyl-L-2,4-diaminobutyric acid (NADA) for 1 h and exposed to 33 µg/mL carbon nanoparticles (CNPs) for further 18 h. Controls were cells incubated with PBS (solvent control) or compatible solutes only. Apoptosis (% hypodiploidy) was determined at 19 h after exposure using propidium iodide staining and flow cytometry. Data are presented as mean ± SEM, (**a**) *n* = 7, (**b**) *n* = 8; * *p* ≤ 0.05.

Besides pro-inflammatory signaling and a reduction in CFTR in lung epithelial cells, carbon nanoparticles are known to decrease apoptosis rates in primed human neutrophils, which are present during inflammation in the lung and under inflammatory conditions in peripheral blood [15]. As neutrophil apoptosis is an important regulator of the life span of this terminally differentiated cell type, this mechanism tightly regulates the strength and duration of inflammation. We therefore determined the amount of apoptotic cells in primed peripheral blood neutrophils 19 h after exposure to an effective dose of 33 µg/mL carbon nanoparticles by estimating hypodiploidy according to Nicoletti (Figure 4a) [35]. From earlier studies we know that, due to high donor to donor variability, high sample numbers are necessary to determine significant effects of particles on blood neutrophils [15]. In our experimental setting with seven volunteer samples, the significant drop in apoptosis rate could only be observed when cells were exposed to carbon nanoparticles alone. In the presence of 1 mM of each compatible solute, respectively, such a reduction in apoptotic cells was not observed. In order to provide a more reliable cell system to study the antiapoptotic effects of poorly soluble nanoparticles, we recently developed a differentiation protocol for the myeloid leukemia cell line HL-60 [32]. With this procedure neutrophil-like cells with specific features of priming and activation similar to inflammatory neutrophils can be produced. Applying this system, we were able to reproduce the effects of carbon nanoparticles in the presence of compatible solutes (Figure 4b). In this experiment the recovery of apoptosis rates by 1 mM of each compatible solute was statistically significant compared with the particles-only exposure.

**Figure 5:**
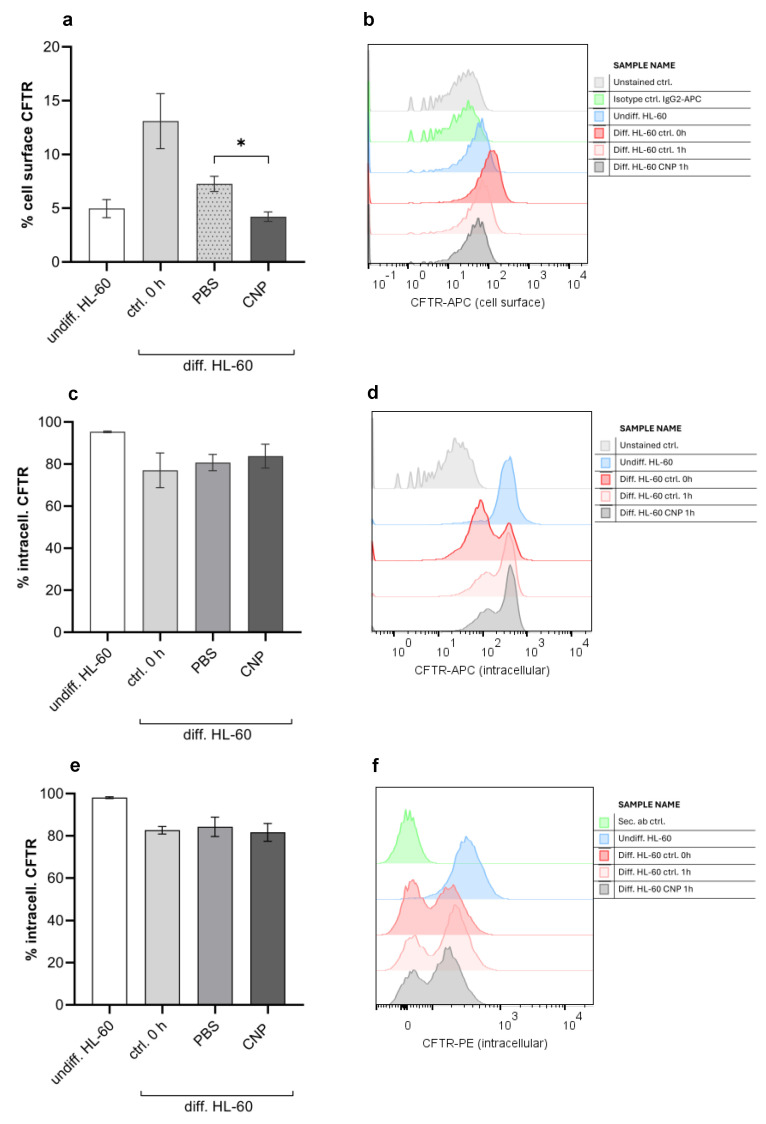
Effect of carbon nanoparticles on CFTR expression in HL-60 cells. HL-60 cells at density of 2 × 10^6^ cells/mL were exposed to 33 µg/mL carbon nanoparticles (CNP) for 1 h. Afterwards cell-surface ((**a**,**b**) with anti-CFTR-APC Ab #NBP2-54509 from Novus Biologicals) and intracellular ((**c**,**d**) with anti-CFTR-APC Ab #NBP2-54509 from Novus Biologicals; (**e**,**f**) with anti-CFTR #Ab-660 Ab from CFTR Antibody Distribution program) expression of CFTR as percentage positive cells were determined by flow cytometry analysis. Controls were undifferentiated HL-60 cells, differentiated HL-60 cells before treatment (ctr. 0 h), or incubated with PBS as a solvent control (ctr. 1 h). (**b**,**d**,**f**) show representative histograms of flow cytometry analysis of CFTR expression. Data are presented as mean ± SEM, (**a**) *n* = 15, (**c**,**e**) *n* = 3; * *p* ≤ 0.05.

In addition to our studies on CFTR expression in lung tissue, we also investigated changes in the membrane channel in neutrophil-like cells in response to carbon nanoparticles and compatible solutes. By using specific antibodies for CFTR in the cytoplasmic membrane and also inside the cell, we determined the amount of this channel by flow cytometry (Figure 5). Fully differentiated HL-60 cells are characterized by an increased amount of CFTR on their surface. After the start of the experiment, this amount was diminished probably due to handling processes. However, after the application of carbon nanoparticles, we observed significantly lower surface CFTR (Figure 5a,b), suggesting that the carbon nanoparticles induce processes leading to a translocation of the chloride channel from the cytoplasmic membrane into the cell. The investigation of intracellular CFTR levels employing two antibodies, which are considered to be specific for this localization, revealed no significant changes after CNP application (Figure 5c–f). Due to the high background of intracellular CFTR, the translocation might not be detectable with this assay.

**Figure 6:**
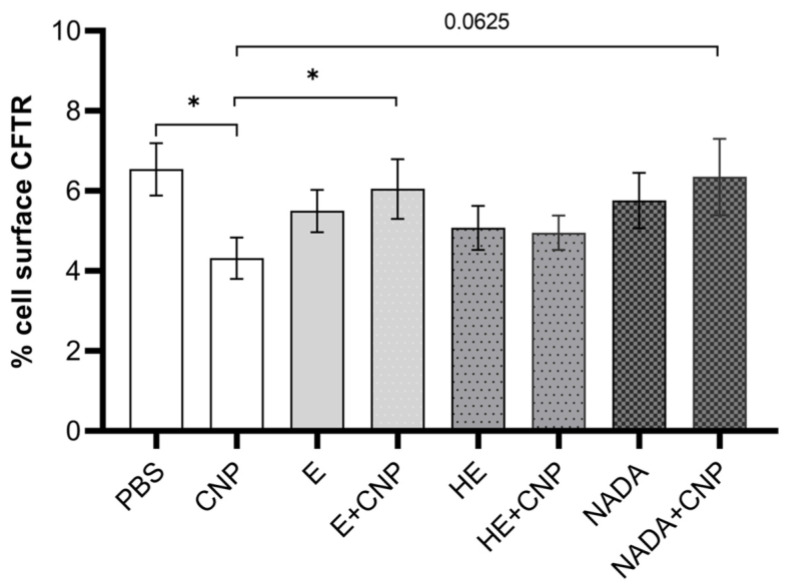
Effect of compatible solutes on carbon-nanoparticle-induced decrease in cell-surface CFTR expression on HL-60 cells. Differentiated neutrophil-like HL-60 cells at density of 2 × 10^6^ cells/mL were pretreated with 1 mM ectoine (E), 5-hydroxyectoine (HE), and Nγ-acetyl-L-2,4-diaminobutyric acid (NADA) for 1 h and exposed to 33 µg/mL carbon nanoparticles (CNPs) for further 1 h. Controls were cells incubated with PBS (solvent control) or compatible solutes only. Cell-surface expression of CFTR (% positive cells) was determined at 2 h after exposure using specific antibody and flow cytometry. Data are presented as mean ± SEM, *n* = 10; * *p* ≤ 0.05.

We therefore concluded that carbon nanoparticle exposure has an impact on the occurrence of CFTR on the surface of neutrophils and possibly contributes to an impairment of the functionality of this cell type. Accordingly, we then aimed to test the effects of these three compatible solutes on the reduction in membrane-coupled CFTR in differentiated HL-60 cells. As the pairwise comparisons show, the decrease in CFTR after carbon nanoparticle exposure cannot be observed when compatible solutes are present during the exposure of differentiated neutrophil-like HL-60 cells (Figure 6). Dunnett’s post hoc testing after ANOVA revealed a significant preventive effect for ectoine and a clear trend for NADA (*p* = 0.06). 5-Hydroxyectoine had considerably smaller effects that did not prove to be statistically significant, probably due to the sample size of *n* = 10.

## 3. Discussion

With our current study, we investigated the effects of three different compatible solutes originating from ectoine/hydroxyectoine metabolism. Effects on lung inflammation were studied by induction of neutrophilic lung inflammation in a mouse model and also by determining apoptosis rates in neutrophilic granulocytes. Our animal experiment clearly showed that all three compatible solutes were able to reduce neutrophil-dominated lung inflammation in the mouse model. The application of the compounds significantly reduced neutrophil numbers in the lung lavage. Additionally, monocytic cell numbers, which are present at a quantity one order of magnitude lower than neutrophils, were reduced. We therefore can conclude that 5-hydroxyectoine and NADA may also be used as strategies for molecular prevention against environmental particulate air pollution. Interestingly, although testing a broad dose range, a clear dose response was not observed for any of the compounds. We therefore have to admit that our intention to rank the substances due to their efficacy failed. The current data suggest a broad range of impact for compatible solutes in the lung. In one of our earlier studies, we were able to show a dose response for ectoine in a rat animal system and in a human bronchial epithelial cell line [12]. As mammals are known to differ in their sensitivity to inhaled poorly soluble particles [36], another system possibly based on human cells might be more useful for the exact evaluation of differences in the efficacy of the substances in reducing lung inflammation induced by environmental particles. Nevertheless, the study is the first to describe the beneficial effects of 5-hydroxyectoine and NADA in this context.

The second aspect of this study on the prevention lung inflammation was to investigate the influence of compatible solutes on the life span of neutrophilic granulocytes after exposure to carbon nanoparticles. We had previously shown that the compatible solutes ectoine and firoin reduce the expression of the antiapoptotic Bcl-2 family protein Mcl-1 and are therefore able to keep apoptosis rates stable after exposure to carbon nanoparticles [16,37]. Our current data show that 5-hydroxyectoine and NADA are also able to counteract the prolongation of neutrophil life span. As the regulation of neutrophil apoptosis is considered as a target to treat chronic inflammation [38], 5-hydroxyectoine and NADA may be considered as additional therapeutics for prevention or treatment of airway inflammation.

A third aspect of this study was to investigate the impact of compatible solutes on the presence of CFTR in cell membranes. We therefore employed our two experimental systems, mouse lung tissue and human neutrophil-like cells, to study this endpoint. Neutrophilic lung inflammation is a typical feature of cystic fibrosis, in which several pro-inflammatory mechanisms in lung epithelial cells and neutrophils are involved [39,40]. Recent findings that spark-generated carbon nanoparticles in human lung epithelial cells (A549) induce a reduction in CFTR suggested that this pathogenic effect also contributes to environmentally induced lung inflammation [22]. Furthermore, individuals suffering from mild forms of cystic fibrosis due to CFTR with moderately reduced functionality may be particularly vulnerable to environmental air pollution. Our results from the animal experiment showing a decrease in CFTR after exposure to combustion-derived carbon nanoparticles, as a proxy of environmental nanoparticles, can be considered as an in vivo confirmation of the earlier in vitro findings. We therefore suggest to investigate this endpoint more closely and evaluate its relevance in the induction of environmentally induced airway diseases. Moreover, data from animals treated with all three compatible solutes show significant effects against the reduction in CFTR. Ectoine, which is already available as a medical device for inhalation, might be considered as a preventive strategy particularly for patients suffering from cystic fibrosis who are exposed to environmental air pollution.

Malfunction or loss of CFTR in neutrophilic granulocytes contributes to the exacerbation of cystic fibrosis after infection [41]. CFTR in neutrophils is mostly found in intracellular phagosome membranes. The neutrophilic pathogen response is therefore impaired in cystic fibrosis patients [42]. The data of this study show that exposure to carbon nanoparticles leads to a decrease in CFTR located in the cytoplasmic membrane. Although we cannot estimate the impact of this effect on impaired pathogen responses by a reduction in the oxidative burst [25], we think that this process should also be considered as possibly pathogenic after an environmental exposure. Moreover, in a second independent cell type, the data corroborate the finding that CFTR is translocated into the cell and not available as a cell-surface chloride channel after exposure to carbon nanoparticles. The finding that all three compatible solutes are able to counteract the decline in CFTR strengthens the hypothesis, which is that a stabilization of functional membrane structures by these compounds is beneficial when humans are exposed to particulate air pollution. The impact of CFTR reductions for human health after environmental exposure and the preventive value of compatible solutes in this context have to be evaluated in human intervention studies. Moreover, the beneficial effects of the compatible solutes ectoine and hydroxyectoine for the treatment of patients suffering from cystic fibrosis have to be investigated in clinical studies. However, promising data from in vitro studies combining ectoine with pharmaceutical CFTR correctors suggest performing such investigations.

## 4. Materials and Methods

### 4.1. Reagents

Particle suspensions were prepared from carbon nanoparticles (carbon black, Printex 90, Degussa AG/Evonik, Frankfurt am Main, Germany) by suspending in PBS as described earlier. Particles and particle suspensions were characterized by transmission electron microscopy and dynamic light scattering [13]. Ectoine ((S)-2-methyl-1,4,5,6-tetrahydropyrimidine-4-carboxylic acid), 5-hydroxyectoine ((4S,5S)-1,4,5,6-tetrahydro-5-hydroxy-2-methyl-4-pyrimidinecarboxylic acid), and NADA, all > 99% LPS-free, were provided by bitop AG (Dortmund, Germany). Solutions were prepared with the indicated concentrations in PBS.

### 4.2. Animal Experiments

Female C57BL/6JRj mice were purchased from Janvier Labs (Le Genet St. Isle, France) at an age of 6 weeks. After two weeks of adaptation, animals (*n* = 7 per group) were subjected to pharyngeal aspiration of particle suspensions or mock controls (PBS) in the absence or presence of compatible solutes as indicated in Figure 1. For that purpose each animal was anesthetized by isoflurane (CP Pharma, Burgdorf, Germany) inhalation (5%) for 2 min. Test suspensions (50 µL) were applied on the pharynx. Shortly after blocking the nostrils, the liquid was inhaled. Animals recovered from anesthesia and exposure within minutes. In order to determine parameters of neutrophil lung inflammation, animals were sacrificed by exsanguination (perfusion with PBS) under deep anesthesia (100 mg/mL ketamine (CP Pharma), 18 mg/kg xylazine (Dechra Pharmaceuticals, Northwich, England)) 12 h after exposure. Lungs were lavaged with 4 × 1 mL PBS. Lung tissue was minced and, after shock-freezing with liquid nitrogen, stored at −80° C until further usage.

### 4.3. Isolation and Cultivation of Human Neutrophils

Human circulating neutrophils were isolated from venous blood by discontinuous Percoll density gradient as described earlier [15]. Isolated cells were suspended in RPMI 1640 medium (Pan Biotech, Aidenbach, Germany) containing 1% FCS (Merck Biochrom, Berlin, Germany), 100 U/mL penicillin/100 µg/mL streptomycin (Sigma-Aldrich, Taufkirchen, Germany), and incubated at 37 °C in a humidified atmosphere with 5% CO_2_.

### 4.4. Cultivation and Differentiation of HL-60 Cells

The human myeloid leukemia HL-60 cell line (wild type CCL-240, ATCC, Manassas, VA, USA) was cultured in RPMI 1640 medium containing 5% FCS (Merck Biochrom) and 100 U/mL penicillin/100 µg/mL streptomycin (Sigma-Aldrich) at 37 °C in a humidified atmosphere with 5% CO_2_. Differentiation into a neutrophil-like state was induced by incubation of HL-60 cells with 1 µM all-trans retinoic acid (ATRA, Sigma-Aldrich) and 1% DMSO (Carl Roth, Karlsruhe, Germany) for 5 days as described earlier [15].

### 4.5. Exposure of Cells

Freshly isolated human neutrophils or HL-60 cells were seeded in 48-well plates at a density of 2 × 10^6^ cells/mL in cell culture medium. Carbon nanoparticle suspension was prepared freshly before use in PBS at stock concentration 1 mg/mL [21]. Stock solutions of compatible solutes ectoine (E), 5-hydroxyectoine (HE) and Nγ-acetyl-L-2,4-diaminobutyric acid (NADA, all from Bitop) were prepared in PBS at concentration 0.1 M and stored at 4 °C until used. Solutes were added to the seeded cells immediately for 1 h prior to particle exposure and solvent control was carried out.

### 4.6. Flow Cytometry of Lavage Samples

Collected broncho-alveolar lavage samples were centrifuged at 250× *g* for 10 min at 4 °C and extracted cells were incubated with erythrocyte lysis buffer prior to blocking in Fc receptor blocking solution (TruStain FcX anti-mouse CD16/CD32 antibody, #101320) according to the instructions of the manufacturer. Immune cell identification was carried out using specific fluorescently conjugated antibodies GR-1-PE (#108407) in a dilution of 1:2000 and CD11c-APC (#117309) in a dilution of 1:800 or the corresponding volume of isotype-matched antibodies PE rat IgG2 (#400307) and APC Armenian hamster IgG (#400911). Blocking solution and antibodies were purchased from BioLegend (San Diego, CA, USA). After the wash step in PBS, cells were stained with 2 µg/mL DAPI (Sigma-Aldrich) as a viability dye prior to flow cytometric analysis with FACSCanto II by use of FACSDiva 6.1.3 software (Becton Dickinson, Franklin Lakes, NJ, USA). After gate-out of residual debris, doublets, and dead cells, the percentages of CD11c^+^ cells representing monocytes/macrophages, GR-1^+^ cells representing neutrophils, and CD11c^-^ GR-1^-^ cells representing lymphocytes were estimated by analysis of the respective dot plot (quadrant statistic). 1 × 10^4^ events per sample were collected and analyzed.

### 4.7. Measurement of Apoptosis

Human neutrophils and HL-60 cells were stained according to Nicoletti protocol by direct DNA staining in propidium iodide (PI, Sigma-Aldrich) hypotonic solution and flow cytometry [35]. PI was dissolved in distilled H_2_O at 1 mg/mL and stored at 4 °C in the dark until used. A total of 6 × 10^5^ cells per sample were suspended in a fluorochrome solution containing 0.1% sodium citrate (*w*/*v*) (Sigma-Aldrich), 0.1% Triton X-100 (*v*/*v*) (Santa Cruz, CA, USA), and 50 µg/mL PI and incubated for 1 h at 4 °C in the dark. Thereafter cells were analyzed by flow cytometry and red fluorescence of PI (>600 nm), bound to the DNA, was measured with FACSCanto II by use of FACSDiva 6.1.3 software (Becton Dickinson). After gate-out of residual debris and doublets, the percentage of hypodiploidy, corresponding to fragmented DNA and thus to apoptotic nuclei, was estimated by analysis of the DNA histogram. A total of 10^4^ events per sample were collected and analyzed.

### 4.8. Flow Cytometry of CFTR

Cell-surface and intracellular CFTR staining were performed in resting and treated HL-60 cells. For analysis of CFTR in resting cells, undifferentiated or differentiated HL-60 cells were immediately used for staining procedure. For CFTR staining in treated HL-60 cells, cell were as first preincubated for 1 h with 1 mM compatible solutes prior to carbon nanoparticle exposure for further 1 h. Fluorescently conjugated human antibody CFTR-APC (#NBP2-54509, Novus Biologicals, Littleton, CO, USA) was used for cell-surface and intracellular staining. Monoclonal unconjugated CFTR antibody provided from Cystic Fibrosis Foundation Therapeutics (#ab660 Chapel Hill, NC, USA) was used for intracellular staining only, as the recognizing domain of CFTR protein (NBD1) is located intracellularly.

Cell-surface staining: First of all, 10^6^ cells per sample were blocked in Fc receptor blocking solution according to the instructions of the manufacturer (human TruStain FcX, #422302 BioLegend) without permeabilization. Afterwards, cells were directly incubated with 10 µg/mL fluorescently conjugated human antibody CFTR-APC (#NBP2-54509 Novus Biologicals) or the corresponding volume of isotype-matched antibody APC mouse IgG2b (#IC0041A R&D Systems, Minneapolis, MN, USA) for 30 min at 4 °C in the dark. Thereafter cells were washed once with PBS, stained with 2 µg/mL DAPI (Sigma-Aldrich) as a viability dye, and analyzed by flow cytometry with FACSCanto II by use of FACSDiva 6.1.3 software (Becton Dickinson). After gate-out of residual debris, doublets, and dead cells, the percentages of CFTR-positive cells were estimated by analysis of the respective histogram (Figure 5b). A total of 2 ×10^4^ events per sample were collected and analyzed.

Intracellular staining: First of all, 10^6^ cells per sample were stained with fixable viability dye eFluor 780 (#65-0865 eBioscience/Thermo Fischer Scientific, Waltham, MA, USA) according to the instructions of the manufacturer for 30 min at 4 °C in the dark. Thereafter, cells were washed once with PBS and fixed in 4% paraformaldehyde (Sigma-Aldrich) in PBS for 10 min at room temperature. After a further wash step in PBS, cells were permeabilized/blocked in Fc receptor blocking solution (#422302 BioLegend) supplemented with 0.1% Triton X-100 (Santa Cruz) for 15 min at room temperature, and afterwards stained with 10 µg/mL fluorescently conjugated human antibody (CFTR-APC #NBP2-54509, Novus Biologicals) for 30 min at 4 °C in the dark. Finally, cells were washed once with PBS before flow cytometric analysis (Figure 5d). Intracellular staining was validated using a second human CFTR antibody. Before staining, 10^6^ cells per sample were stained with fixable viability dye and permeabilized/blocked as described above. Thereafter cells were stained with unconjugated monoclonal mouse anti-human CFTR antibody (CFTR #Ab-660, Cystic Fibrosis Foundation Therapeutics—CFTR Antibody Distribution program) in a dilution of 1:500 overnight at 4 °C. The stained cells were washed in PBS and stained with a secondary PE-conjugated (Fab’)_2_ goat anti-mouse IgG H+L antibody (#A10543 Thermo Fischer Scientific, Waltham, MA, USA) in a dilution of 1:500 for 1 h at room temperature in the dark. Finally, cells were washed once with PBS before flow cytometric analysis (Figure 5f).

Graphics for publication were created by use of FlowJo 10.8.1 software (Becton Dickinson).

### 4.9. Protein Isolation and Western Blot

Proteins were isolated in RIPA buffer (Cell Signaling, Danvers, MA, USA) from frozen tissue samples containing inhibitors for protease and phosphatase (Roche, Basel, Switzerland) employing a ball mill at 2500 rpm for 2 × 35 s. Protein concentration was determined in the supernatant after centrifugation at 16000× *g* for 10 min at 4 °C using a BCA protein assay kit (Pierce^TM^, Thermo Fischer Scientific). Proteins (25 µg per slot) were separated on 4–15% polyacrylamide gradient gels (Bio-Rad, Hercules, CA, USA). Blotting on PVDF membrane (Bio-Rad) was performed under wet blot conditions at 100 V for 2 h at 4 °C in Tris glycine buffer (24 mM Tris-base, 183 mM glycine, and 20% methanol, all from Sigma-Aldrich). After blocking for 1 h with TBST containing 5% milk powder (Carl Roth), membranes were incubated overnight at 4 °C with primary antibodies CFTR A-3 (#sc-376683 Santa Cruz, Dallas, TX, USA) in a dilution of 1:500 in TBST + 5% BSA (Carl Roth), and GAPDH 6C5 (#ab8245 Abcam, Cambridge, England) in a dilution of 1:10,000 in TBST + 5% BSA. Secondary anti-mouse HRP-conjugated antibody (#7075 Cell Signaling) was added in TBST + 5% milk powder for 1 h at room temperature. Luminescence was induced by using ECL Prime (Cytiva Amersham^TM^, Marlborough, MA, USA) and visualized with the Chemi Premium Imager (VWR, Radnor, PA, USA). Band intensities were quantified using the Image Capture Software 1.6.6.0 (VWR). Band intensities of CFTR band were normalized to GAPDH as a housekeeper.

### 4.10. Statistical Analyses

Analyses were performed with GraphPad Prism version 9.4.1 for Windows (GraphPad Software, Boston, MA, USA). Data analyses of the animal experiment (Figure 2) were performed applying two-way ANOVA with a mixed-effects model, after correction for outliers. Post hoc analyses (Dunnett’s) were performed in order to determine differences between groups. Multiple comparisons were performed by applying one-way ANOVA with Dunnett’s post hoc tests for statistical significance. For the analyses of significant differences between two groups, ranked tests were applied (Wilcoxon matched-pairs test or Mann–Whitney ranked test). Data were considered to be statistically significant when *p* ≤ 0.05.

## Data Availability

The datasets used and/or analyzed during the current study are available from the corresponding author on reasonable request.

## Supplementary Materials

The following supporting information can be downloaded at: https://www.mdpi.com/article/10.3390/ijms26199487/s1.

## Abbreviations

Ab	antibody
APC	allophycocyanin
ATCC	American Type Culture Collection
ATRA	all-trans retinoic acid
BCA	bicinchoninic acid
Bcl-2	B cell lymphoma-2
BSA	bovine serum albumin
CD	cluster of differentiation
CFTR	cystic fibrosis transmembrane conductance regulator
CNPs	carbon nanoparticles
COPD	chronic obstructive pulmonary disease
DAPI	4′,6-diamidin-2-phenylindol
DMSO	dimethyl sulfoxide
DNA	deoxyribonucleic acid
E	ectoine
EGFR	epidermal growth factor receptor
FACS	fluorescent-activated cell sorting
FCS	fetal calf serum
GAPDH	glycerinaldehyd-3-phosphat-dehydrogenase
HE	hydroxyectoine
HRP	horseradish peroxidase
Ig	immunoglobulin
LPS	lipopolysaccharide
MAP	mitogen-activated protein
Mcl-1	myeloid cell leukemia-1
NADA	Nγ-acetyl-L2,4-diaminobutyric acid
PBS	phosphate buffered saline
PE	phycoerythrin
PI	propidium iodide
PVDF	polyvinylidene difluoride
RIPA	radioimmunoprecipitation assay
RPMI	Roswell Park Memorial Institute
SEM	standard error of the mean
TBST	tris-buffered saline with Tween20
UV	ultraviolet
