# Supplementary material for: Compatible Solutes Prevent Lung Inflammation and Reduction in CFTR Induced by Combustion-Derived Nanoparticles in Human and Rodent Experimental Systems
Source: Int J Mol Sci. 2025 Sep 28;26(19):9487. doi: 10.3390/ijms26199487 (PMC12524578; doi:10.3390/ijms26199487)
Supplement: Supplementary file 1 [file ijms-26-09487-s001.zip › ijms-3807622-supplementary.pdf]

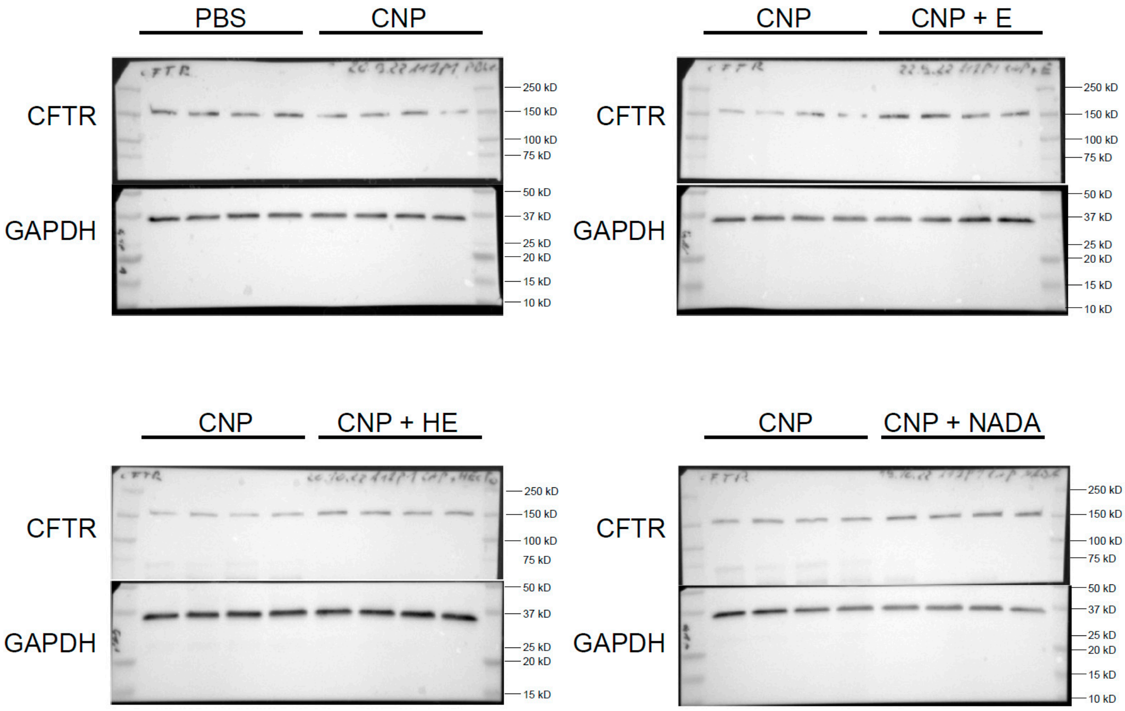

Figure S1. Effect of compatible solutes on carbon nanoparticle induced decrease of CFTR in the lung (uncropped blots). Female C57BL/6JRj mice were exposed via pharyngeal aspiration to 5 mg/kg carbon nanoparticles (CNP) in the presence or absence of 1 mM ectoine, 5-hydroxyectoine (HE), and Nγ-acetyl-L-2,4-diaminobutyric acid (NADA). Protein expression was analyzed in lung homogenates using specific antibody for Western blotting. Membranes were cut after blotting for separate incubation with antibodies against cystic fibrosis transmembrane conductance regulator (CFTR) and glyceraldehyd3-3-phosphate-dehydrogenase (GAPDH).
